# Molecularly Imprinted Polymers for Ochratoxin A Extraction and Analysis

**DOI:** 10.3390/toxins2061536

**Published:** 2010-06-18

**Authors:** Jorn C. C. Yu, Edward P. C. Lai

**Affiliations:** 1 College of Criminal Justice, Sam Houston State University, Box 2525, 1003 Bowers Blvd., Huntsville, TX 77341, USA; 2 Department of Chemistry, Ottawa-Carleton Chemistry Institute, Carleton University, Ottawa, ON, K1S 5B6, Canada; Email: edward_lai@carleton.ca

**Keywords:** molecularly imprinted polymer, ochratoxin, solid phase extraction, analytical method, review

## Abstract

Molecularly imprinted polymers (MIPs) are considered as polymeric materials that mimic the functionality of antibodies. MIPs have been utilized for a wide variety of applications in chromatography, solid phase extraction, immunoassays, and sensor recognition. In this article, recent advances of MIPs for the extraction and analysis of ochratoxins are discussed. Selection of functional monomers to bind ochratoxin A (OTA) with high affinities, optimization of extraction procedures, and limitations of MIPs are compared from different reports. The most relevant examples in the literature are described to clearly show how useful these materials are. Strategies on MIP preparation and schemes of analytical methods are also reviewed in order to suggest the next step that would make better use of MIPs in the field of ochratoxin research. The review ends by outlining the remaining issues and impediments.

## 1. Introduction

In an attempt to explain the enzyme–substrate interactions, the lock-and-key mechanism was originally proposed by Nobel laureate Emil Fischer back in the 1970s. Although this theory was later disproved by X-ray diffraction studies, the idea of a matrix-designed material that recognizes a particular substrate remained the cornerstone of molecular imprinting [1]. Over decades of research, molecularly imprinted polymers (MIPs) have gained more and more acceptance with respect to their application as polymeric antibodies in analytical chemistry and separation science [2,3,4]. MIPs are solid materials that can be synthesized via a molecular imprinting process, in which a template molecule (the target compound) is present during polymerization. Thus, template molecules are imprinted into the polymer via the molecular imprinting process. After the removal of the template from the polymer, the end-product polymer is called a MIP, in which specific binding cavities with a shape and functional groups complementary to the template are created within the polymer matrix [5]. Due to the presence of specific binding cavities in MIPs, they exhibit specific selectivity for the template molecule, or group selectivity for a class of structurally related molecules. Thus, MIPs mimic the function of natural antibodies (monoclonal or polyclonal) [6,7]. They often afford high adsorption capacities (~50 mg/g), good site accessibilities, fast binding kinetics (~10 min), and good recoveries (~90%). Many applications using MIPs have been reported, such as the removal of 2,4-dichlorophenol from contaminated water [8], the sensing of amino acids via a quartz-crystal microbalance (QCM) [9], the detection of dansylated amino acids via a surface plasmon resonance sensor [10], the sensing of zearalenone with fluorescent probes [11], the detection of caffeine with molecularly imprinted quantum dot photoluminescence [12], the detection of amino acids via a capacitive sensor [13], food analysis [14,15,16], and the recognition of the tobacco mosaic virus [17], just to name a few. The application of MIPs as sorbents allows not only pre-concentration and cleanup of the sample, but also selective extraction of the target analyte, which is particularly important when the sample matrix (such as environmental and biological samples) is complex and impurities can interfere with quantification [18].

Mycotoxins are secondary metabolites that molds produce naturally. Ochratoxin A (OTA) is a carcinogenic mycotoxin of wide natural abundance, as produced by several species of *Aspergillus* (e.g., *A. ochraceus*) and *Penicillium* (e.g., *P. verrucosum*) fungi [19,20,21]. It is suspected to cause the Balkan Endemic Nephropathy, which is a fatal kidney disease observed in rural areas of southeast Europe [22]. OTA has been found as a contaminant in food and feed and exhibits multiple toxicities in animals and mankind, including nephrotoxic, hepatotoxic, immunotoxic, teratogenic, and carcinogenic effects, which represent serious health risks to livestock and the general population [23]. The widespread occurrence of OTA in cereals, wheat [24], maize, rice, beans, nuts, raisins, and beverages (such as milk, coffee, grape juice [25,26], and wine [27,28,29]) has prompted health regulation authorities to define maximal tolerable daily intake levels (5 ng/kg body weight) [30]. Epidemiology studies in Bulgaria, Romania, Spain, the Czech Republic, Turkey, Italy, Egypt, Algeria, and Tunisia have also found significantly higher serum or plasma levels of OTA in patients with certain kidney disorders compared to healthy people, although the association may not be a causal one [31]. Due to their ubiquitous presence in foodstuffs and their potential risk for human health, prompt detection is deemed important. An excellent review on analytical methods for the determination of mycotoxins has recently been published by Turner and co-workers [32]. The development of analytical methods for OTA identification generally involved liquid–liquid extraction, clean-up by an immunoaffinity column (IAC), and identification by HPLC with fluorescence detection (HPLC-FLD) [33]. Various extraction and clean-up procedures for the determination of OTA by HPLC-FD in musts, wine, and beer were compared: (1) dilution with polyethylene glycol 8000 and NaHCO_3_ solution and clean-up on an IAC; (2) extraction with chloroform and IAC clean-up; (3) solid phase extraction (SPE) on C18; (4) SPE on reverse-phase phenylsilane; and (5) SPE on Oasis HLB cartridges [34]. The former IAC procedure was simple, rapid, and provided flat baselines that were free from most impurity peaks, high OTA recoveries and quite repeatable results. Unfortunately, the IACs are relatively costly and have a short shelf life. For the assay of real samples (cereals, oat, corn, *etc.*), denaturation of antibodies by the organic co-solvent needed to dissolve hydrophobic analytes in aqueous solution is a serious problem. Thus, the design and the synthesis of biomimetic antibodies that can specifically bind a target molecule has long been a research goal [35]. The use of MIPs as artificial antibodies for sample pretreatment was described, first in a review on emerging sorbent materials for SPE [36], and next in a report on immuno-based sample preparation for trace analysis [37]. Over the next few years, MIPs have increasingly attracted attention as substitutes for immunoanalysis (e.g., binding assays, biosensors, and solid-phase immune-extraction) [38]. The development of a new sorbent for selective SPE that is capable of OTA preconcentration prior to HPLC analysis is an important issue. An affordable SPE sorbent, compared to the use of IAC columns, will make the screening of foodstuff more frequent in both developed and poor countries, thus protecting human health [39]. An overview of conventional and emerging analytical methods for the determination of mycotoxins is written by Cigić and Prosen [40]. 

Although molecular imprinting has been around for over 30 years, recently, this technology has made rapid developments. Due to the variety of structures of mycotoxins, it is possible to use the molecular imprinting technique for their analysis and selective detection. As early as 2004, a two-dimensional extraction procedure employed solid phase extraction (SPE) and MIP for the extraction of OTA [41]. Here direct sample loading onto the MIP resulted in low recoveries, thus prior removal of interfering acidic matrix compounds by C18 SPE was deemed necessary. Unfortunately, a similar result was observed in a control experiment, in which the MIP was replaced by the corresponding non-imprinted polymer (NIP). These findings suggested that specific binding to molecularly imprinted sites played a minor role in OTA enrichment. Recent developments in imprinting technology have made possible the practical application of MIPs in mycotoxin detection. The structure activity relationships of reported MIPs for OTA, deoxynivalenol (DON), zearalenone (ZEA), and moniliformin (MON) have been reviewed by Appell, Maragos, and Kendra [42]. MIPs offer quite a promising tool for the future development of antibody-based methods, in which compatibility between sample extract and antibody is a major limiting factor. The current trend in OTA determination is largely towards the powerful combination of liquid chromatography with tandem mass spectroscopy (LC-MS/MS) [43]. An emerging focus of LC-MS/MS is in the field of multi-residue methods for the simultaneous determination of mycotoxins that are being considered by the EU legislation in force (*i.e.*, aflatoxins, fumonisins, ochratoxin A, trichothecenes, and zearalenone). This topic has been investigated by several research groups, although major problems with extraction and cleanup steps have not been fully resolved [44]. LC-MS/MS is a powerful tool for the identification and quantification of masked mycotoxins, which are mycotoxins conjugated to more polar compounds (e.g., glucose) that are not detected by routine analytical methods.

## 2. Sensor Development for Ochratoxins Using MIPs as Sensing Elements

Although most validated detection methods are chromatographic, alternative strategies based on biosensing principles are emerging. The factors which worked against unattended, continuous monitoring of toxins included the inherent instability of suitable protein receptors and antibodies, and the irreversible nature of the binding event (which necessitates a continuous supply of reagents for sequential measurements). Nevertheless, biosensors were hopefully capable of being used for on-site measurement of contamination by specific toxins. Methods for improving the stability, extending the range, and for altering the binding characteristics of sensing molecules would be essential, as discussed previously by Paddle [45]. An evaluation of MIP films for coulometry used an applied positive potential to induce adsorption of the target molecules [46]. The resultant sensors showed a high degree of sensitivity, selectivity, and a broad linear range. Imprinting a polymer matrix with binding sites located at the surface has been shown to be advantageous for use as the sensor interface. The binding sites are more accessible, the mass transfer is faster, and the binding kinetics are faster [47]. A molecularly imprinted polypyrrole (MIPPy) film was synthesized on the Spreeta sensor, a miniaturized surface plasmon resonance (SPR) device, for the detection of OTA [48]. The MIPPy was electrochemically polymerized on the sensor surface from a solution of pyrrole and OTA in ethanol/water (1:9 v/v). The film growth was monitored *in situ* by an increasing SPR angle. The binding properties of the MIPPy film were investigated by loading OTA standard solutions into the integrated 20-μL flow cell. After 300 s, nonlinear regression was used to determine the maximum binding signal. Spreeta results showed that the signal was measurable for OTA concentrations down to 0.05 ppm. Pulsed elution with 1% acetic acid in methanol/water (1:9 v/v) was found to be efficient for the regeneration of the MIPPy film surface. Interference by the matrices of wheat and wine extracts was evaluated. No significant binding of the wheat extract with MIPPy was observed when acetonitrile/water (1:1 v/v) was used as the mobile phase. Biosensors and sensor arrays provided selective, sensitive, and accurate measurements. The feasibility of miniaturizing biosensors and sensor arrays, so that they are portable, makes them useful as screening bio-tools meant to ensure the correct assessment of mycotoxins in food so as to reassure the consumer [49]. The interfacing of a suitable transducer to MIPs is still growing and is expected to have a more significant impact in the field of biochemical sensors. A rapid and highly sensitive SPR assay of OTA has recently been reported, using Au nanoparticles for signal enhancement on a mixed, self-assembled monolayer surface, in a competitive immunoassay format [50]. Although an enormous effort is being put into developing biosensors, relatively few toxic analytes can yet be measured by commercially available devices.

## 3. Molecularly Imprinted Solid Phase Extraction (MISPE) for Ochratoxins

### 3.1. Selection of Functional Monomer

To make good MIPs, the selection of suitable functional monomers, cross-linkers, porogen solvents, initiators, and polymerization procedures require careful consideration [51]. Crucial to the success of these efforts is the rational design of novel basic and neutral functional monomers, so as to allow the maximization of the template-functional monomer association via ion-pairing, hydrophobic, and steric interactions. Due to the complexity of such factors as functional monomer-template complexation, solvent effect, and cross-linking density that drive the imprinting process, the performance of any new MIP towards the target molecule is rather difficult to predict. The development of MIP for a specific application still relies on empirical optimization. The specificity of a MIP is governed by the factors mentioned above (at the preparation stage) and by experimental conditions at the binding stage. Many vinyl monomers and different cross-linkers (polyfunctional acrylics) are available commercially at a low cost [52]. Free radical polymerization is usually the method of choice for preparing MIPs [53]. To induce radical polymerization, an appropriate quantity of initiator is required. Macroscopic polymer networks have been most widely synthesized. These MIPs tend to be insoluble materials that provide rigidity and mechanical stability to all imprinted binding sites. Since most MIPs are prepared in the form of a macroporous monolith, the grinding and sieving process is required to yield proper particle sizes for analytical applications. This preparation of MIPs inherits some drawbacks, such as intensive labor, insufficient yield, and potential exposure to hazardous airborne particles when toxic molecules are imprinted.

Chen *et al.* observed enthalpic changes attributed to the rebinding of template molecules to the MIP by micro-calorimetric studies [54]. The results suggest that a single one-point interaction is insufficient to induce selectivity, regardless of the strength of this interaction. Selectivity requires molecular recognition based on multiple electrostatic interactions and secondary interactions, such as hydrophobicity and macroscopic phase separation. Spivak *et al.* have determined that shape selectivity is an important contributing factor to the overall MIP selectivity [55]. It was found that branched-structure templates produce higher-selectivity MIPs than their straight-chain counterparts. Shape selectivity, as determined by steric exclusion or optimal fit, maximizes binding interactions.

With the development of computer technology and quantum chemistry, the computational study of MIPs has emerged in hopes of making a good selection of functional monomers that maximize the molecular recognition property of MIPs. Wulff *et al.* used the electrostatic potential surface obtained by MolCad to indicate the shapes of occupied and unoccupied molecular cavities of MIP [56]. There was a report on using molecular modeling software to study the functional monomer-template conformation before polymerization [57]. Chianella *et al.* employed a virtual library of functional monomers to screen against the target template molecule, and the selectivity of MIP was greatly improved [58,59]. These results suggested that computer modeling of MIP synthesis, analysis, and evaluation would be a promising method for the fast, accurate, safe, and economical study of MIPs. Recently, a theoretical and experimental study of nicotinamide MIPs with different porogens was conducted by Wu *et al.* [60]. Good correlations have been found between the interaction energy and the selectivity. When the porogen had poor hydrogen bonding, the interaction energy was mainly influenced by the dielectric constant of the solvent. When the porogen had a strong capacity in forming the hydrogen bond, both the dielectric constant and the hydrogen bonding would affect the formation of the template–monomer complex. This computational study strongly suggested that the interaction energy between the template and monomer varied with the pre-polymerization solution composition. When using aprotic solvents (such as chloroform, toluene and acetonitrile), the interaction energies are mainly influenced by their dielectric constants. The smaller the dielectric constant is, the stronger the complexation between template and monomer will be. When using solvents (such as methanol) with a high capacity to form hydrogen bonds, the hydrogen bonding would affect the formation of the template-monomer complex, thus influencing the interaction energy. This successful prediction model may provide a better way of selecting a good solvent for a given a template and functional monomer system.

Preparation of MIPs for OTA recognition is challenging, as relatively few reports can be found in the literature. They are all listed in Table 1. It is noteworthy that dual function monomers were sometimes employed to improve specific recognition of individual OTA template molecules, by better shape complementarity of the polymeric binding pocket and by two specific types of electrostatic interactions.

**Table 1:** Functional monomers used for OTA MIP preparation from the literature.

Functional Monomer	Structure	Reference
*N*-Phenylacrylamide	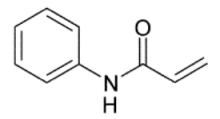	[61]
A mixture of methacrylic acid acrylamide(dual functional monomers)	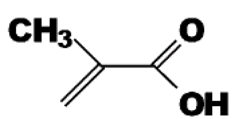 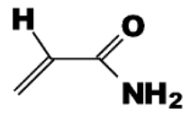	[62]
methacrylic acid	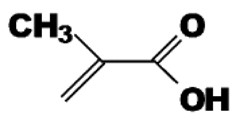	[63]
pyrrole	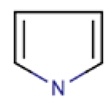	[64,65,66]
A mixture of diethylaminoethylmethacrylate (DEAEM) and itaconic acid (IA) (dual functional monomers)	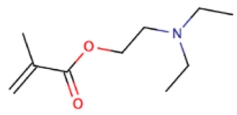 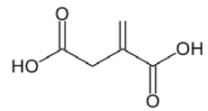	[67]

Molecularly imprinted polypyrrole (MIPPy) for OTA recognition via electrochemical imprinting has been shown to be promising [68,69]. Easy preparation was expected with the new approach. Furthermore, it has been revealed by N1s X-ray photoelectron spectroscopy (XPS) studies that the hydrogen of N-H on the pyrrole ring within PPy undergoes protonation in acidic conditions. Under alkaline conditions, the N-H will be deprotonated [70]. The removal of the OTA template molecules from MIPPy could be achieved by perturbing the hydrogen bonding between the OTA analyte and the MIPPy binding cavity. Uptake of the target analyte would be enhanced when the N-H on the pyrrole ring is protonated.

### 3.2. Optimization of Extraction and Desorption Procedure

From the practical understanding of MIP development, a number of rules of thumb have emerged in the literature that are helpful when developing particular MIPs [71]. It is generally believed that the quantity and quality of MIP recognition sites are related to the mechanisms and strengths of the monomer-template interactions present in the pre-polymerization mixture. The general guideline, “the more stable or stronger the monomer-template complex is, the more selective the MIP will be,” has been presumably used as a rule in the selection of functional monomers [72]. As for the selection of porogen solvents, it is generally accepted that the MIPs synthesized with aprotic porogens usually have higher affinity and selectivity than those prepared using porogens with moderate or strong hydrogen bonding capacity [73]. These general guides were drawn from the conventional preparation of MIPs, which were prepared by using methacrylic acid (MAA) as a functional monomer, ethylene glycol dimethacrylate (EGDMA) as a cross-linker, and an aprotic solvent as the porogen. Thus, hydrogen bonding and electrostatic interactions could operate strongly in the non-aqueous environments. In aqueous environments, hydrogen bonding and electrostatic interactions could be disrupted. Hydrophobic interactions, which are non-specific, could govern analyte retention. An ideal MIP is generally the result of good spatial orientation and intermolecular interactions (such as electrostatic, hydrogen bonding, and hydrophobic interactions) in both non-aqueous and aqueous environments. The recognition affinity of MIPs is increased when they are synthesized in a high-concentration template solution [74]. Scatchard analysis often reveals that a binding site of heterogeneity does exist [75]. In SPE procedures, various parameters affecting the selectivity of the extraction can be optimized [76]. The MIP is then washed with an appropriate solvent that is capable of disrupting the non-specific interactions of analytes with the polymer matrix. In a practical analytical procedure, pulsed elution has been developed by Mullett *et al.* to eliminate non-specific bindings [77]. 

### 3.3. Molecularly Imprinted Micro Solid Phase Extraction and Limitations

In pharmaceutical, clinical, environmental and food chemistry applications, solid-phase extraction (SPE) has been widely employed for the isolation and pre-concentration of target analytes, as well as for the clean-up of sample matrices [78]. However, matrix interference components can be co-extracted with the target analytes when conventional SPE sorbents, such as C18, ion-exchange, and size-exclusion materials, are used. The co-extraction and co-elution problems produce undesirable results. For example, detector signals can be suppressed or enhanced when matrix components are co-eluted with the analytes. These effects are particularly problematic in the case of trace analysis.

Many affinity-based sorbent materials have been developed to selectively extract the target analytes, such as boronate, lectin, protein A or G, synthetic dyes, immobilized metal ions, aptamers, peptides, antibodies, and molecularly imprinted polymers (MIPs) [79,80]. Among these affinity techniques, both antibodies and MIPs can be used as single analyte- or group-selective sorbents. Particles of an MIP material can be either packed into a micro-column [81,82,83] for selective SPE, or glued to the surface of a solid-phase micro-extraction (SPME) fiber [84]. New fabrication techniques continue to be developed for molecularly imprinted solid phase extraction (MISPE) and molecularly imprinted solid phase micro-extraction (MISPME) [85].

In the framework of research and development, some problems inherent to MISPE were previously addressed. These included the reproducible preparation of MIP sorbent materials with consistent molecular recognition characteristics, the risk of non-specific bindings, the potential for repeated use of MIP, unfavorable polymer swelling in application-relevant solvents, potential sample contamination by template bleeding, and slow analyte binding kinetics. Several attempts have been proposing new alternatives to minimize the inherent drawbacks of the preparation and use of MIPs. Most notably, Tamayo *et al*. gave an overview on the significant attempts carried out during recent years to improve the performance of MIPs in solid-phase extraction [86]. Leakage of template during the storage of MIP materials has been reported [88]. A good laboratory practice should resolve this concern. Template mimic seems to be an alternative solution to resolving this limitation of MISPE [87].

### 3.4. Strategies on the Preparation of MIP

Techniques for bead formation have been investigated to obtain more homogeneous MIP particles within a narrow size range [88,89]. Precipitation or emulsion polymerization has gained attention due to better control of particle sizes and morphologies [90]. However, these types of polymerization usually require either the use of special dispersing phases or complicated swelling processes [91]. Molecular imprinting to make MIP nanomaterials has gained more and more attention since 2005 [92]. MIP nanomaterials can be synthesized by precipitation or emulsion polymerization, to be readily suspended in aqueous media. Ye *et al.* have prepared MIP nanomaterials with controllable size in the nano- to micro-meter range [93]. Varying the composition of the cross-linking monomer allowed the particle size to be altered in the range from 130 nm to 2.4 μm, whereas the favorable binding property remained intact. Applying MIP onto gold nanoparticles for sensing has been shown to be a promising strategy [94]. Pavel and co-workers used computational tools not only to investigate the monomer-template interaction, but also to simulate the template-MIP interaction [95,96]. They used molecular dynamics simulation to predict the interaction energies, the closest approach distances, and the active groups for different bio-ligands. They found that electrostatic interactions play the most significant role in the formation of MIP materials. Acrylic acid, methacrylic acid, acrylamide, acrolein, acrylonitrile, styrene, *etc.*, (a total of 25 monomers) were simulated. The minimized structures of five ligands (theophylline, theobromine, theophylline-8-butanoic acid, caffeine, and theophylline-7-acetic acid) were obtained with the use of the molecular mechanics approach. The simulation results indicated that ligands interact with either-COOH or [CH_2_=CH]- functional groups at the MIP surface. It was also found that a molecular structure without functional side groups can be used for molecular imprinting. With several successes in MIP computational modeling, some experimental parameters do not seem fully compatible with regard to the reality. These parameters do not account for effects, such as the disruption of monomer-template complexes by the cross-linker, the modulation of monomers activity as they are increasingly incorporated into the growing polymer network, and the influence of the porogen solvent.

### 3.5. Schemes of Analytical Methods

The possibility of solvent-dependent tuning of substrate selectivity or affinity, and the high binding capacity, render MIPs as promising SPE adsorbents for pre-concentration of OTA from various biologically relevant matrices [97]. Solvent effect on the recognition properties of MIP specific for OTA was reported by Turner *et al.* [98]. Application of multivariate analysis to the screening of new MIPs was proposed before [99]. The effect of temperature on OTA biosorption onto yeast cell wall derivatives was studied to elucidate isotherms and thermodynamic parameters [100]. Carbon nanotubes (CNTs) were used successfully to enhance the binding capacity of a molecularly imprinted polypyrrole (MIPPy)-modified stainless steel frit for micro-SPE to determine OTA in red wines [82]. Elution of OTA from MIPPy/CNTs, for on-line coupling to HPLC analysis with fluorescence detection, was achieved by an ion-pairing mechanism using 2% triethylamine. For a 3 mL sample of red wine, the limit of detection was 0.08 ppb (S/N = 3), which is more than adequate for handling the regulatory level of 2 ppb.

### 3.6. Preparation of MIP Micro-Particles and Nano-Particles

Three different formats of imprinted polymers can be prepared via bulk, precipitation, and emulsion polymerization methods [101]. The synthesis of MIP microspheres, using MAA and triallyl isocyanurate, was reported as early as 2005 [102]. A simple surface molecular imprinting technique was developed to synthesize MIP-coated SiO_2_ micro-particles in aqueous solutions [103]. They were demonstrated to have high adsorption capacity, excellent selectivity, and site accessibility for the target organic pollutant.

Soluble MIP nanoparticles were synthesized via iniferter initiated polymerization and separated by size via gel permeation chromatography [104]. Subsequent fractionation of these particles by affinity chromatography allowed the separation of high-affinity fractions from the mixture of nanoparticles. Fractions selected this way possess affinity similar to that of natural antibodies (Kd = 6.6 × 10^-8^ M) and were also able to discriminate between related functional analogs of the template.

## 4. MISPE for LC-MS/MS Analysis of Ochratoxins and Metabolites

Among xenobiotics, applications of MS techniques for the analysis of toxins, pesticides, drug residues, amines, and migrants from packaging were previously overviewed [105]. OTA in grape was determined by nano-HPLC coupled with ESI-mass spectrometry [106]. Currently, there is a trend towards developing multi-mycotoxin methods for the simultaneous analysis of several Fusarium mycotoxins belonging to different chemical families that is best achieved by LC-MS/MS (liquid chromatography with tandem mass spectrometry). Krska *et al.* gave an overview of the commonly used methodology for the analysis of fumonisins (FBs), moniliformin (MON), zearalenone (ZON), and trichothecenes in feeds [107]. Good limits of detection for OTA in wine samples (1.3–3.4 μg/kg) by LC/MS/MS have also been demonstrated by Reinsch *et al.* [108]. In order to reduce the ion suppression effect on MS/MS analysis, the use of MISPE for sample clean-up will be a promising development in the future. 

## 5. Molecularly Imprinted Solid Phase Extraction (MISPE) for Other Mycotoxins

The major mycotoxin-producing fungi are species of *Aspergillus*, *Fusarium*, and *Penicillium* and the important mycotoxins are aflatoxins, fumonisins, ochratoxins, cyclopiazonic acid, deoxynivalenol/nivalenol, patulin, and zearalenone [109]. In 2006, MIP Technologies signed an exclusive distribution agreement with Supelco [110]. SPE cartridges that were prepared using MIP technology were termed SupelMIP SPE by the company. This commercialization of MIP materials offered tailor-made selectivity for the extraction of trace analytes in complex matrixes. The MISPE products provided faster sample preparation and better MS compatibility (reduced ion suppression), allowing analysts to achieve lower detection limits and improved sensitivity. By the end of 2009, there were 20 different SupelMIP SPE cartridges available in the market. Most target analytes were pesticides and drugs. Unfortunately, no SupelMIP SPE cartridge was made for ochratoxins. However, consultation on the preparation of new SPE phases is available from the company. In the future, development and research for other specific SupelMIP SPE cartridges will be expected.

## 6. Remaining Issues and Impediments

To prepare MIPs with good recognition properties toward target compounds, proper selection of functional monomers has always been the first task for most researchers. The complexity of multiple interactions among the pre-polymerization components makes the selection of functional monomers still a big challenge. A simplified computational approach is often employed to develop a prediction of the resulting MIP. Predictive modeling of functional monomer interactions and the resulting binding site performance provides a more rational design of synthetic strategies for new MIPs. It can be expected that upon establishment of more complex models that provide better resemblance of the actual MIP preparation conditions, computational modeling will develop into a useful complementary tool, efficiently reducing the large number of MIPs that are currently screened to achieve optimal recognition properties. However, the accuracy of predictive simulations will strongly depend on the complexity of the model and on experimental conditions. Hopefully, refined simulations, along with supporting spectroscopic data, would lead to more accurate modeling of pre-polymerization complexes and binding sites. Spectroscopic studies can gather evidence for self-assembly based on ionic and π–π stacking interactions (rather than previously assumed hydrogen bridge bonding), leading to a refined model of the pre-polymerization complex [111]. In Table 1, a functional monomer with high affinity towards OTA, a mix of functional monomers, and a novel conductive polymer, are all feasible for the preparation of MIPs for OTA extraction and detection.

Direct imprinting of OTA was found to be not ideal for the preparation of OTA MIPs [69] due to its slow release of OTA template molecules. Structure analogs, such as *N*-(4-chloro-1-hydroxy-2-naphthoylamido)-(L)-phenylalanine, have been used as a surrogate in the preparation of MIPs for OTA extraction [80]. New desorption chemistry (such as electrochemical, electrospray desorption) with fast kinetics and efficiency for template removal should be investigated in the future. Ideally, slow release of template can be eliminated to prevent any potential carry-overs. As for the recovery of OTA from different samples matrices, possible binding of OTA to proteins or other sample matrix components was tested by acid treatment before extraction, but no significant differences with controls appeared [112]. It is in the best interest of researchers to find new ways for the ease of MIP preparation, ease of OTA removal from MIP, and ease of use for real-world samples. These remaining issues will definitely need a broad investigation of novel polymer chemistry.

## 7. Conclusions

MIPs are synthetic receptors with high-affinity sites that can selectively recognize a target analyte, based on its shape, size, or functional group distribution. These receptors are promising due to their easy preparation, thermal stability, chemical inertness, and long shelf life at room temperature and humidity. From the point of view of analytical chemistry, this protocol is very promising for applications in the extraction and analysis of ochratoxins. Recent investigations have led to the synthesis of new MIPs for a wider range of mycotoxins. Currently, MIPs are not selective enough in the aqueous environment to compete with natural antibodies [113], and better shape selectivity must be achieved in future development. In addition, mycotoxins are costly for the large-scale preparation of MIPs. The concept of template mimics, surrogate templates, or dummy templates must be used to prepare MIPs that are both affordable and selective for the target mycotoxins [105,114]. A couple of peptide receptors have recently been selected for ochratoxin A using computational methods by screening *de novo* designed peptide libraries. Affinity characterization resulted in K_A_ = 63 mM^-1^ for the 13-mer peptide and K_A_ = 84 mM^-1^ for the 8-mer peptide [115]. Last, researchers must be able to determine a more proper wash solution to remove OTA template molecules from the strongest-binding MIP cavities, especially in ultra-trace analysis (by LC-MS/MS).
